# The cost of cancer: a retrospective analysis of the financial impact of cancer on young adults

**DOI:** 10.1002/cam4.657

**Published:** 2016-02-07

**Authors:** Michelle S. Landwehr, Samantha E. Watson, Catherine F. Macpherson, Katherine A. Novak, Rebecca H. Johnson

**Affiliations:** ^1^ The SamfundBostonMassachusetts02111; ^2^ Children's Hospital Los AngelesLos AngelesCalifornia; ^3^ Mary Bridge Children's Hospital/MultiCareSeattleWashington

**Keywords:** Cancer, cost of illness, financial support, health expenditures, young adult

## Abstract

Young adult cancer survivors (YAs) are confronted with immense financial challenges in the wake of their treatment. Medical bills and loss of savings may cause YAs to forgo recommended medications or follow‐up appointments. Young survivors with financial concerns also report depression, stress and anxiety. The Samfund is a national nonprofit organization that provides financial support to YAs post‐treatment. To quantify the financial burden of cancer in YAs, a retrospective analysis was performed of data collected from Samfund grant applications of 334 YA cancer survivors. Grants were awarded between 2007 and 2013 and grant recipients were consented electronically in 2014 for retrospective data analysis. Recipients ranged from 19 to 39 years of age at the time of their grant applications. Descriptive statistics were calculated and compared to the Medical Expenditure Panel Survey (MEPS) and U.S. census data on age‐matched peers. Financial indicators of YA cancer survivors are worse in many domains than those of age‐matched controls. Furthermore, YA survivors in their 30s report more perilous prefunding financial situations than younger grant recipients. Cancer has a devastating and age‐specific impact on the finances of YAs. Philanthropic grants from the cancer support community, in conjunction with healthcare policy reforms, have the potential to break the cycle of financial need and help YAs move forward with their lives after cancer treatment.

## Introduction

Cancer is a more expensive illness than most people realize 1. In addition to the obvious costs, including medical bills and prescription co‐pays, financial status is negatively impacted by missed work, costs of follow‐up care and a paucity of financial assistance resources, particularly for patients who have completed cancer therapy 1, 2, 3. The financial burden of cancer is uniquely challenging for young adults (YAs), who lack not only the life experience and financial security of older adults but also the parental support that may benefit children. As a result, YAs may finish treatment with empty savings accounts and bleak financial futures. Patients experiencing financial hardship may struggle with impossible decisions about which bills to pay and which to skip, whether to risk going without insurance or medications or declare bankruptcy instead 2, and determining if and how to pay for follow‐up care 4, 5. These challenges may engender feelings of depression and anxiety, and may negatively impact quality of life 6.

The Samfund, a national nonprofit organization, has provided direct financial assistance and online support since 2005 to help YAs reduce debt, live independently and move forward toward their goals. Data collected by The Samfund over the past decade provide a detailed representation of the experiences of that subset of YA survivors who have faced severe financial toxicity 7, 8 and negative economic consequences as a result of their diagnosis and treatment.

### Cancer in YAs

Approximately 70,000 YAs aged 15–39 were diagnosed with cancer in the United States in 2011 9. Cancer is the leading cause of disease‐related death among YAs, and over the past 20 years survival rates for YAs have improved much less than other age groups. This disparity is thought to relate to inferior clinical trial enrollment and lack of research focus on this age group 10, 11. Due to population growth and increasing cancer incidence, however, the number of YAs in the United States is expected to grow over the next decade 10, 11, 12. Many will live for decades, often coping with lingering medical and financial effects of cancer treatment.

### Cost of cancer treatment

Cancer treatment in the United States is extremely expensive and costs continue to escalate 1. Particularly concerning are the inferior health outcomes noted in economically disadvantaged individuals – dangerous in a population with preexisting healthcare needs 13, 14. Furthermore, medical debt is a common cause of bankruptcy, especially in cancer patients 2. In one study, adult cancer patients filed for bankruptcy 2.65 times more frequently than age‐matched peers without cancer 2. However, few studies have commented on the financial health of YAs. One report noted that bankruptcy occurs most frequently in cancers that predominate in YAs compared to older adults, including lymphoma, leukemia, and thyroid cancer 3. YAs who attempt fertility preservation incur significant additional expenses, since insurance rarely covers procedures or storage 15.

### Financial issues specific to YAs

YAs are the most likely of all age groups to be uninsured, dramatically increasing the potential financial burden of cancer treatment 16, 17. Among adults, YAs are the age group at greatest risk for unemployment:^18^ 12.4% of YAs aged 20–24 years and 7.4% of those aged 25–34 years are unemployed 18. YAs are less likely to be employed than their siblings or peers without a cancer history 17. Half of full‐time students and workers with cancer endorse difficulty continuing with school or work 17, 19. More than one third of YAs report that cancer negatively impacted their long‐term educational or career plans 1.

Financial problems among YAs may result in decreased access to health care 4, 20. Obtaining insurance can be a tricky process even for healthy YAs, as they transition from school to the workforce and from parents' coverage to individual plans. Coverage among young adults 19–34 has increased since the Affordable Care Act (ACA) and state Medicaid expansions, though affordability still remains one of the largest challenges to enrollment 21. Patients without health insurance are at risk for inconsistent follow‐up care 4 and negative health outcomes. YAs are more likely than elderly survivors to report financial barriers to medical care 4, 5, and are also 56–67% more likely than age‐matched peers to forego medical care due to its cost 5.

Over one third of all cancer survivors in the U.S. report unmet needs for financial assistance during treatment 22. Far less is known about financial problems of cancer survivors following therapy 20. Some report that their financial difficulties are “almost worse than the disease itself 22.” Better supportive care to address financial concerns has the potential to improve both physical and mental health outcomes for YA survivors 22.

## Methods

### Sample

Data for this analysis were obtained retrospectively using deidentified information from grant applications submitted to The Samfund between 2007 and 2013. The Samfund awarded approximately 100–120 grants annually per year during this period (579 grants total), averaging $1,676.96 per award. Eligible applicants resided in the Unites States and were cancer survivors aged 17–35 years until 2012, and aged 21–39 in 2013 and after. Survivors were defined as patients free of cancer, >1 year following completion of cancer therapy, or in remission on maintenance therapy.

The study sample contains data from applicants who received grants between 2007 and 2013. In 2014, all screened applicants from that time period (*n* = 1,102) were contacted via email to request use of their data for this study. Of those contacted, 710 did not respond, 21 refused, and 371 (33.6%) electronically consented to analysis of their deidentified data. Most of those who consented were funded applicants (72%) with a slightly higher mean age at application ([consenters: mean age = 28.7 years, median age = 29 years, 95% CI [−2.0, −0.5], SD = 4.6 years), (refusers: mean age = 27.4, median age = 28 years, 95% CI, SD = 5.1 years]) at *P *< 0.01. Ultimately only funded applicants were included, a total of 334 participants (57.7% of all eligible funded applicants). Age of funded applicants ranged from 19–39 in this sample. In Figure 2, an age‐matched subsample (25–34) of 194 was used for comparison.

### Measures

Applications of funded grant recipients who consented for the study were retrospectively analyzed. Applicants provide both demographic information and essays. In Part I of the application, survivors report their age, state of residence, living situation, data regarding cancer diagnosis and treatment dates, and extensive financial information regarding income, expenses, assets and liabilities (debts). Part II includes multiple essays, depending on specific funding requests and whether the applicant was funded previously. Applicants describe their cancer experience, its impact on finances, the immediacy of need, other sources of financial support, and how a grant would help them pursue life goals. Applicants submit a medical history verification signed by a clinician, a medical records release, and tax documentation from the past two years. A volunteer panel composed of cancer community partners, former grant recipients, and other volunteers with a keen understanding of the challenges YAs face, reviews the applications using established measures. Selection criteria include severity of financial need, lack of other sources of support, and articulation of how a grant will help applicants move forward with their goals. The panel members review each application individually and subsequently meet as a group to rate them. The panel then makes funding recommendations which are reviewed and approved by The Samfund Board of Trustees. At 6, 12 and 24 months post‐funding, recipients are asked to rate their educational, professional and living situations and provide qualitative updates in essay form, describing changes in their lives and financial situations.

### Analysis

Because no control group was available for this analysis, U.S. census data from 2011 and 2013 (using the groups “under age 35” and “25–34 years of age”, respectively) 23, 24, 25 and the Medical Expenditure Panel Survey (MEPS) (using the group “18–44 years of age”) 26 were utilized as proxies for comparison data. Census reports vary: the 2011 reports used displayed household debt and net worth, whereas the 2013 reports included income and poverty data. The MEPS was chosen because it reports healthcare spending in the general population, as well as information about employment, income, demographics, and health care usage. These weighted samples were deemed acceptable proxies for control data due to the similar age range and the robust size of each dataset, though the datasets were different enough that further statistical comparisons were not conducted.

Within the Samfund study sample, financial indicators were compared between recipients under 30 years of age versus 30–39 years of age, using independent samples (two‐way *t*‐tests). Financial indicators analyzed included credit card debt, total liabilities, monthly income and expenses, and monthly medical and student loan expenses. To more precisely define how the financial struggles of YA cancer survivors vary by age, the study sample was also divided into quintiles and compared financial indices using *t*‐tests. The relationship between homeownership and age was assessed via Pearson's Chi‐squared test.

## Results

### Financial status of Samfund grant recipients

Age in the study sample ranged from 19 to 39 at the time of application submission (mean age = 29.3 years, median age = 30.0 years, 95% CI [28.7, 29.8], SD = 4.4 years). Gender breakdown was 79.6% female, and 20.4% male, representative of The Samfund general applicant pool. Recipients resided in 42 of the 50 United States, and had completed cancer therapy a mean of 3.5 years prior to submission of their applications (median = 1.8 years, 95% CI [3.0, 4.0], SD = 4.6). Their mean age of most recent diagnosis was 24.5 years (median = 26 years, 95% CI [23.7, 25.2], SD = 6.7) with an average of 1.2 cancer diagnoses per person (293 of the 334 listed one cancer diagnosis, 33 listed two, five listed three, and three listed four). Samfund grant awards ranged from $200 to $10,000. Recipients were funded a mean amount of $1,707.43 (median = $1,500.00, 95% CI [$1,603.76, $1,811.11], SD = $961.74).

**Figure 1:**
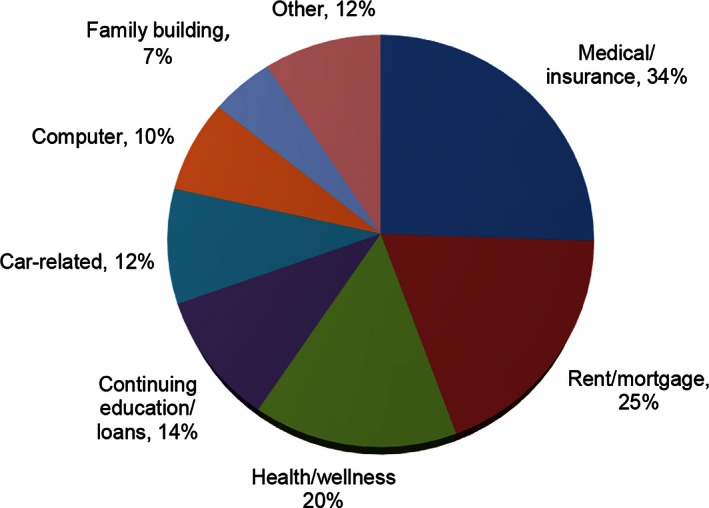
This pie chart reflects the categories of funding for Samfund grants distributed to YAs between 2007–2013. Medical/Insurance, Rent/Mortgage, and Health/Wellness were the most commonly awarded categories.

The categories of funded grants are shown in Figure 1. Applicants may make up to three requests and are typically funded for one or two. “Medical/insurance” grants include payments on current or residual medical bills, insurance premiums, prescription co‐pays, dental expenses, or cosmetic/reconstruction procedures. “Rent/mortgage” includes living expenses and utilities. “Health/wellness” pertains to physical therapy, mental health payments, gym memberships, yoga, or alternative healing modalities including acupuncture and massage therapy. “Continuing education” refers to tuition, certificate program or testing fees, and books, as well as student loan payments. “Car‐related” grants include car payments, repairs, or insurance. “Family building” grants fund banking, freezing and storage of sperm, oocytes or embryos, procedures such as IUI or IVF, or adoption‐related expenses. Grants in the “other” category include, but are not limited to: public transportation, professional apparel, groceries, and home modifications.

### Comparison with U.S. census data and medical expenditure panel survey (MEPS)

**Figure 2:**
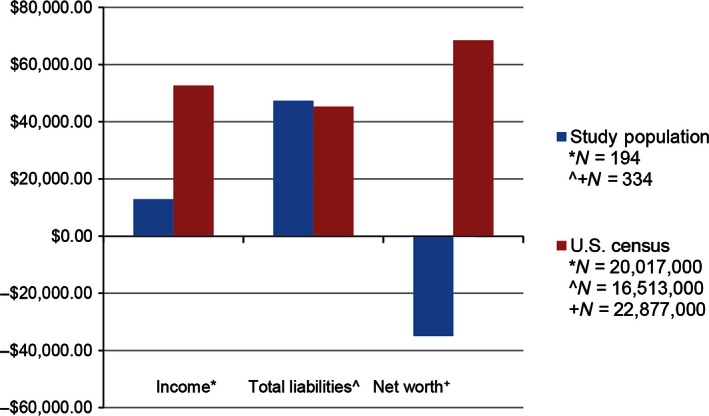
This bar graph illustrates the various levels of disparity in income, total liabilities, and net worth between the study population (YAs between 19–39) and their approximately age‐matched householders from U.S. Census Data.

Figure 2, which illustrates discrepancies in income, liabilities and net worth, compares groups within the study sample (*N* = 194 or 334) to proxy data for groups of comparable age the “average” population from the U.S. Census in 2011 and 2013 23, 24, 25. The age groups in the U.S. Census that most closely matched our study sample were “18–35 years of age,” in the 2011 census (*N* = 16,513,000) and “15–35 years of age” (*N* = 21,877,000) in the 2013 census, both of which are slightly younger groups that Samfund grant recipients, who ranged from 19–39 years of age. To compare income of Samfund grant recipients to that of controls in the general population, data were extracted from the corresponding similar age range in the U.S. census. The largest differences between the study group and the general population occurred in the area of net worth (defined as the value of all things owned by an individual, such as home, car, cash, plus any investments, less any debts). According to U.S. Census data, the mean net worth of YAs is $68,479.00 in assets 25. In contrast, YAs applying for Samfund grants had mean negative net worth of –$35,009.41 in debts. No further statistical analysis was pursued because the Samfund, U.S. census and MEPS datasets and age ranges were different, albeit grossly comparable.

Table 1 displays key financial indices in The Samfund's study sample, comparing YA cancer survivors aged 19–29 years to those aged 30–39 years at the time of their grant application. We assessed total liabilities, including student loans, medical and credit card debt, unpaid taxes, bank loans, mortgage balances, loans on retirement accounts, or other debts such as personal loans. Second, we evaluated mean annual net income from all sources. Among the 68 YAs under age 30 receiving supplemental income (which may include SSI/SSDI, food stamps, or child support), the mean amount was $1,355.75 (median = $825.00, 95% CI [−$547.65,$256.05], SD = $1,615.55), significantly less (*P* = 0.03) than the 61 YAs over age 30 receiving supplemental income, for whom the mean amount was $1,903.40 (median = $1,600.00, SD = $1,244.11). Some of the most pronounced financial differences in the age groups occur in the category of total liabilities and monthly expenses, both of which are significantly larger for the older group. Mean total liabilities were $37,760.16 in the younger group versus $59,012.16 in the older group. Monthly expenses averaged $1,490.94 for the younger group versus $2,135.70 for the older group (median = $1,395.00 and $2,143.00 respectively, 95% CI [‐$947.13,‐$342.37], SD = $1,127.04 and $1,585.26 respectively) and differed significantly at *P *< 0.01. There was less of a pronounced difference in monthly income ($1,385.84 vs. $1,851.14), though still also statistically significant at *P* = 0.04 (median = $680.00 and $1,780.00 respectively, 95% CI [‐$906.56,‐$24.05], SD = $2,326.31 and $1,627.11, respectively). The greater liabilities in the older group may be due to in part to credit card debt. On average, participants in the younger group reported $3,025.93 in total credit card debt whereas the older group reported $3,913.89, although this comparison was not statistically significant. YAs over 30 were also more likely to own a home. In YAs under 30, 36.6% (*N* = 49) reported having a mortgage, compared to 37.7% of YAs over 30 (*N* = 57). A chi‐square test was performed and a significant relationship was found between age and home ownership, *χ* ^*2*^ (1,*N* = 334) =4.60, *P *= 0.032.

**Table 1:** Financial indices of Samfund grant recipients (19–39 years of age) at the time of initial grant application (2007–2013)

	All *(N = *334*)*	19–29 (*N *= 183)	30–39 (*N *= 151)
Mean total liabilities^a^	$47,368.10	$37,760.16	$59,012.16
Mean total medical debt	$3,898.29	$3,616.89	$4,239.34
Mean total credit card debt	$3,427.37	$3,025.93	$3,913.89
Mean monthly income^a^	$1,596.20	$1,385.84	$1,851.14
Mean monthly expenses^b^	$1,782.44	$1,490.94	$2,135.70
Mean monthly medical expenses	$210.73	$184.25	$242.82
Mean monthly student loan payment	$92.54	$112.35	$68.53
Mean income to expenses ratio	0.88	0.87	0.89

a *P *< 0.05 for under 30 versus 30+ groups.

b *P *< 0.01 for under 30 versus 30+ groups.

In Table 2, the impact of age on financial status was analyzed by comparing financial indices between different age groups of grant recipients at the time of their grant applications. The study sample was divided into quintiles: 19–21, 22–25, 26–30, 31–35, and 36–39 years of age. Liabilities were highest in the group aged 31–35 years. Total medical debt was lowest in the youngest recipient group. Credit card debt was highest in groups aged 30–39. Finally, the income‐to‐expense ratio was assessed, which indicates a person's likelihood of being able to pay their bills each month. A score of 1.0 or higher indicates ability to meet expenses. Income‐to‐expense ratio was lowest (most unfavorable) in the youngest group, who are more likely to be in high school or college and therefore generating less income. In the study sample, only the group aged 22–25 years showed evidence of being able to meet their expenses from month to month. All other age groups of Samfund grant recipients, on average, had scores of less than 1.0 and therefore had expenses that exceeded their incomes. Furthermore, out‐of‐pocket medical expenses among Samfund grant recipients averaged $2,528.76 annually (*N* = 334) (Table 2), markedly higher than the median $610.00 reported by 18–44 year‐olds surveyed by the MEPS in 2013 (*N* = 111,957,000) 26.

**Table 2:** Financial indices of Samfund grant recipients (19–39 years of age who received grants between 2007 and 2013) at the time of initial grant application by age quintiles

	All *(N = *334*)*	19–21 (*N *= 11)	22–25 (*N *= 51)	26–30 (*N *= 121)	31–35 (*N *= 119)	36–39 (*N *= 32)
Mean total liabilities^a^^,^ d	$47,368.10	$17,496.67	$29,421.78	$43,116.82	$60,972.47	$51,722.25
Mean total medical debt	$3,898.29	$1,479.00	$3,216.41	$3,980.33	$4,565.80	$3,025.29
Mean total credit card debt^c^^,^ d ^,^ e	$3,427.37	$1,381.64	$1,286.33	$3,908.64	$3,784.05	$4,396.74
Mean monthly income^f^	$1,596.20	$2,350.27	$1,107.55	$1,415.46	$1,839.89	$1,892.99
Mean monthly expenses^a^^,^ c ^,^ d ^,^ e ^,^ f ^,^ g	$1,782.44	$1,500.28	$1,136.73	$1,639.40	$2,071.35	$2,374.98
Mean monthly medical expenses	$210.73	$83.27	$135.40	$214.01	$217.28	$337.77
Mean monthly student loan payment	$92.54	$175.73	$77.47	$121.29	$71.15	$58.82
Mean income to expenses ratio^a^^,^ b	0.88	0.34	1.08	0.84	0.90	0.86

a *P *< 0.05 (19–21 vs. 31–35).

b *P *< 0.05 (19–21 vs. 36–39).

c *P *< 0.05 (22–25 vs. 26–30).

d *P *< 0.05 (22–25 vs. 31–35).

e *P *< 0.05 (22–25 vs. 36–39).

f *P *< 0.05 (26–30 vs. 31–35).

g *P *< 0.05 (26–30 vs. 36–39).

In their initial application essays, Samfund grant recipients reported negative financial sequelae related to cancer in statements such as, “When I became sick, my working hours were reduced. The resulting financial decline forced me to rely on my credit card. Each month, I pay the minimum amount due, but the finance charges essentially negate my payments. The mounting debt terrifies me. It will permanently affect my credit, thus impacting my life forever (female, age 29).” Similarly, essays indicated stress induced by medical debt: “There's just nothing left after I've paid my normal person bills of rent, car payments, grocery bills, school loans, etc., so the bills have accumulated. The hospital calls regularly to urge me to pay more. I always pay what I can, but that leaves me with nothing to help re‐build my savings account, to visit friends, to buy a pair of shoes or get ahead (female, age 30).” Lastly, one YA recounted an inability to maintain follow‐up care: “I am not able to go to my PT [physical therapy], primary care or radiation oncology visits…because I can't have yet another expense (female, age 34).” Fourteen of 104 YAs in the sample (13.5%) mentioned foregoing medical follow‐up appointments or skipping/reducing their medications as a result of financial strain.

Grant recipients provided qualitative and quantitative data in follow‐up surveys 6, 12, and 24 months following grant funding. Anecdotally, many respondents reported decreased stress and increased hope and empowerment in their follow‐up surveys. One recipient noted, “[The grant] allowed me to continue to pay for my health insurance during a time that I didn't know where the money was going to come from. It relieved a lot of the financial stress… and allowed me to focus on my schoolwork instead of…my health care costs (female, age 32).” Another reported, “[Receiving a Samfund grant] gave me confidence. After feeling so out of control of my life from cancer, it empowered me. I now have the resources to get my life back on track (female, age 29).”

## Discussion

The YA survivors in this study face substantial financial challenges. They have markedly lower median income, greater out‐of‐pocket expenses, and lower net worth than YAs of similar age who responded to the 2011 and 2013 U.S. Census 23. According to the Commonwealth Fund, 36% of YAs between 19 and 29 years of age in the general population report having medical debt 27, whereas 51% of the Samfund study sample reports having medical debt. YAs who leave work during cancer treatment may lose important professional development opportunities. Unlike healthy peers who have worked without interruption, these survivors must later contend with gaps on their résumés when applying for jobs. YAs lack the seniority and job security that accrues with time in many fields and may ease re‐entry into the workforce for older cancer survivors. Anecdotally, Samfund recipients reported losing their jobs and subsequently having great difficulty being re‐hired, sometimes because they are still too ill to work full time. YAs face lost work productivity related to cancer treatment, which may impact subsequent career advancement 18, 28.

The sample data suggest that the financial impact of cancer is more severe in YAs aged 30–39 years compared to those under age 30. Although older YAs may be more to likely to marry and receive additional income through a partner, they may receive less parental assistance and have greater likelihood of dependent children and homeownership than the younger group. These facts could account for the observation that credit card debt is more than twice as high in YAs aged 26–39 compared to YAs under age 25. Medical debt in this sample increased consistently between 19–21 and 31–35 years of age. Medical debt rose most sharply in survivors aged 26–30 years in comparison to younger YAs aged 22–25 years, a 23.8% increase. Medical expenses also increased with age and were four times as high in the oldest compared to the youngest quintile of YA survivors in this dataset (Table 2).

Financial hardship may result in medical nonadherence 14, 29, which has a measurable impact on outcomes and is more marked in YAs than in older adult cancer survivors 4, 5, 29. Numerous studies have documented that physicians are unlikely to initiate discussions about finances with adult cancer patients and survivors 6, 8, 14, 30. The 13.5% in the sample who reported foregoing care is likely an underreporting of the true frequency, because the Samfund application and follow‐up essays do not explicitly ask about medical nonadherence. Broad, public recognition of financial struggle in cancer survivors, along with empowerment of patients to discuss financial issues openly with their healthcare providers, are important first steps toward controlling the cost of cancer therapy in the Unites States.

Finally, to date the medical literature has focused on the negative psychosocial impact of cancer‐related financial hardship 6, 7, 31, 32. Future studies should explore whether the presence of adequate financial support is associated with progression toward psychosocial recovery following cancer therapy. Furthermore, as a result of the Affordable Care Act (ACA) and state Medicaid expansion, 5.7 million young adults who previously had no health insurance have obtained coverage since the first open enrollment period in October 2013 33. Future exploration of pre‐ versus post‐ACA financial burden in the YA cancer population will also be interesting to consider, though no research examining long‐term financial impacts of this legislation has yet been conducted.

### Study limitations

Selection bias is a limitation of this study, as this is a purposive sample composed of individuals with self‐reported financial difficulties related to cancer. It appears that in this sample, cancer has a more negative financial impact on YAs aged 30–39 than on younger YAs, women are overrepresented, and the areas of greatest financial need are medical bills, insurance, living expenses, health and wellness endeavors, and continuing education or student loan payments. Since The Samfund has grown over the past decade and data were collected for internal purposes, certain demographic and financial characteristics were not assessed from the start of initial data collection (e.g., racial/ethnic identification has never been collected at the time of application to prevent reviewer bias, nor has education level; partner's income and expenses were added a few years into data collection). Though financial indices did not differ substantially between funded and non‐funded applicants, those who received grants may also have been more likely to consent to participation in this study. Future studies with a nationally representative sample of YAs are urgently indicated, in order to further define the financial needs of YA survivors, the characteristics of patients seeking financial assistance, and effective interventions to help YAs overcome financial toxicity.

## Implications

Within the healthcare system, multidisciplinary oncology providers should discuss finances openly with YA survivors. This dialog has the potential to empower YAs, a population at‐risk for adverse financial, psychosocial, and health outcomes 12, and steer them toward reputable sources of financial support. Numerous not‐for‐profit agencies target the YA population and provide financial aid, education and guidance. Grants such as those from The Samfund provide immediate financial relief by helping YA survivors pay expenses and/or debts, and may also offer renewed hope and confidence to grant recipients.

Further studies should examine the factors that promote successful financial recovery in YA cancer survivors and help them move forward in their lives.

## Conflict of Interest

Authors have no conflict of interest disclosures to report.
